# Dynamiceuticals: The Next Stage in Personalized Medicine

**DOI:** 10.3389/fnins.2017.00329

**Published:** 2017-06-07

**Authors:** Jose L. Perez Velazquez

**Affiliations:** Neuroscience and Mental Health Program, Division of Neurology, Department of Paediatrics, Institute of Medical Science, The Hospital for Sick Children, University of TorontoToronto, ON, Canada

**Keywords:** personalized medicine, deep brain stimulation, dynamical systems, machine learning, translational medicine, neuromodulation, epilepsy

## Abstract

The surge in the interest in personalized medicine necessitates a corresponding rational approach for implementing such individualized therapies. Dynamiceuticals represents a natural extension of the Pharmaceutical and Electroceutical fields, where the precise determination of the dynamical regimes of the pathophysiology will guide to devise therapies that ameliorate the pathology in a well-controlled manner, thus being precisely tailored toward the implementation of individualized medicine. This approach foretells to lessen side-effects and achieve superior efficacy as compared with current trial-and-error or open-loop strategies. But does the current state of knowledge and technology allow this scheme to offer what it claims?

## Introduction

The great rise in neurostimulation procedures, being applied to a wide variety of syndromes (epilepsy, Parkinson's, addiction, pain) and other settings (e.g., cognitive enhancement), generates the essential need for an “intelligent” approach, for it is being recognized the lack of knowledge guiding the stimulation paradigms and about the effects of the stimulation. Some recent texts (Bourzac, 2016; Eisenstein, 2016) have mentioned, albeit superficially, the need to find out how neurostimulation works in order to avoid risky side-effects and to increase the efficacy. The nature of the problem is best exemplified in the words of a neurologist at the University of Oxford investigating deep brain stimulation (DBS) in Parkinson's; talking about the results of DBS on dysfunctional motor-circuit rhythms, he states: “You can impede them by timing the stimulation appropriately, or you can worsen them by timing it wrong” (Eisenstein, 2016). Thus, considering our existing knowledge of brain dynamics and the frameworks already in place to scrutinize physiological dynamics, added to the development of “personalized medicine,” time seems propitious for the emergence of a new field. It can be called *Dynamiceuticals*, presaged by the advent of Electroceuticals that arose as a substitute for Pharmaceuticals when medications are not efficacious, difficult to administer or cause insidious side-effects; one further step takes us to the concept of Dynamiceuticals: the understanding and use of the system's own dynamics to control, ameliorate pathological conditions. As such it can be considered an “intelligent” electroceuticals or pharmaceuticals—for one can use any of these two approaches within the more general field.

Either using pharmacotherapy or electrotherapy, most of approaches presently done to treat patients are blind to the intrinsic dynamics of the organ or system in question. Thus, detailed knowledge of the dynamical regimes of the tissue responsible for the syndrome is rarely employed as guiding principles in the clinical implementation of the therapies. The most typical is a trial-and-error strategy, such as it is normally done in current DBS paradigms, which ends up in a fixed-frequency stimulation (periodic pacing) by means of stimulating devices operating under open-loop control (open-loop protocols are therapies delivered according to pre-programmed schedules that are not influenced by changes in the dynamics of the underlying syndrome). This theme is not restricted to neuroscience only, although it is this field, witnessing a fast development in devices—especially the relentless miniaturization trend of electronic instruments—added to the adverse side-effects of medications in the treatment of neuropsychiatric syndromes, that will determine the necessity for the dynamiceutical approach. The text that follows is, inevitably, a personal perspective not entirely free of prejudice.

## A dynamic personalized medicine

Science normally advances with small steps. It took a long time to realize that not all people's physiology and biochemistry are created equal. While there was not too much weight on dynamical thoughts at the stage at which healthcare practitioners and scholars started contemplating the possibilities of individualized therapies, the notion of “personalized medicine” spread relatively fast. This occurred despite the fact that it entails a shift in what is the customary focus of attention of scientists and clinicians: the average. Here we talk about evaluating the responses to interventions in the individual, not in the grand average from the whole population. The departure from the glorified averages, mean values, standard errors and the like will still take time to fully permeate the healthcare community, but we are getting there. Originally driven by genetics, currently the concept of personalized medicine is extending its horizons and thus one finds articles commenting on the need for customizing neurostimulation technology.

And yet, the definition is still centered on statics, be it genetic analysis or responses to drugs, with a hint toward the dynamics (in italics in the definition below). Take for instance a look at the European Commission Research and Innovation website where one can find how the Horizon 2020 Advisory Group has defined personalized medicine: “a medical model using characterization of individuals' phenotypes and genotypes (e.g., molecular profiling, medical imaging) for tailoring the right therapeutic strategy for the right person at the right time, and/or to determine the predisposition to disease and/or to *deliver timely and targeted prevention*.” The dynamiceutical approach consists in characterizing the dynamical regimes of biological mechanisms in pathologies—and in normal physiology, for understanding the normal sheds light on the abnormal. It applies to neurostimulation in epilepsy, to circadian rhythm sleep–wake disorders, to reproductive disorders…To everything where one or several variables can be measured to obtain a time series, the standard manner that dynamical system theory functions in order to characterize the system's dynamical regimes. Consider, as an illustration, the studies performed time ago on the hormonal control of reproduction to treat reproductive disorders; treatment with pulsatile injections of hormones like GnRH, by means of pumps programmed at adequate frequencies, led to restoration of ovulation (Reid et al., 1981). It applies as well to the search for anticancer compounds that arrest mitosis, as the dynamiceutical approach can help resolve the recognized challenge by Chan et al.: “the unpredictable complexities of the human body's response to these drugs still herald the biggest challenge toward clinical success” (Chan et al., 2012). Indeed, the dynamics of mitotic oscillators have been classically studied using stability diagrams of mitotic oscillations based on certain enzymes' rates. Critical care medicine is a fertile ground for this approach as well; most of physiologic support therapies such as mechanical ventilators or drug infusions are performed at constant rate, and there is evidence that these protocols are not the optimal for recovery from critical states. Current advances implement variability in the administration protocols, like the neurally adjusted mechanical ventilation (Schmidt et al., 2010). These new strategies implementing variability in medical treatments represent a course toward the use of the real understanding of the intrinsic dynamics in the design of the therapy.

Perhaps more crucially, I foresee a most fundamental application for the (near) future in psychiatry. In fact, notions like biotypes or neurotypes are being advanced: “…we must learn to identify each individual patient's ‘computational neurotype,’ recognizing that people with similar symptoms may have quite different neural system impairments and thus quite different pathophysiologic processes and treatment needs” (Vinogradov, 2017). As we can see, the dynamic perspective finds boundless applications that transcend physiological systems and pathological conditions.

## A dynamiceutical illustration: similar approach to brain and cardiac control

As remarked above, the benefits of dynamiceuticals are not restricted to the brain, but apply equally to other systems, like the heart, e.g., for the control of cardiac arrhythmias (Hall et al., 1997; Christini et al., 2001). It is thus expedient to consider the similarities in the control of cardiac rhythms and that of brain rhythms, to comprehend how a similar basic approach can be applied to very different pathologies and organs. A recent study reports the efficient control of epileptiform activity in rat models of seizures using a protocol for intracerebral stimulation (Salam et al., 2015) that was derived from previous detailed scrutiny of the brain synchronization dynamics in epilepsy. In both cases a similar analytical approach was performed: the examination of the possible steady states, or fixed points of the dynamics and their stability. The brain studies started using simplistic *in vitro* seizure models (Khosravani et al., 2003) and continued with analysis of *in vivo* recordings in rats and patients (Perez Velazquez et al., 2003). The essential observation in the heart and brain studies is that specific fixed points of the dynamics were found using very similar analytical methods: state space reconstruction of the dynamics using time delay plots of either atrio-ventricular conduction intervals, in the case of the heart, or the intervals between peaks found in the neurophysiological recording. The cardiac study used this geometric approach to stabilize a cardiac rhythm that became unstable in arrhythmia (Christini et al., 2001). The brain study sought to stabilize a steady state that did not lead to the paroxysmal discharge typical of the seizures (Khosravani et al., 2003). Other fundamental observations in the brain study included the change in the synchronization between brain networks preceding seizures, event that finds usefulness in detecting a possible upcoming paroxysm and hence can be employed in a feedback (also termed closed-loop, or on-demand) DBS protocol. As opposed to the abovementioned open-loop protocols, closed-loop stimulation strategies only stimulate when specific patterns or alterations in the (neuro)physiology in question are detected by the system. While this is not the place to dwell into details (readers interested in the specific comparison between the two approaches are referred to Perez Velazquez and Frantseva (2011)), the point to be appreciated is that, at this level of description, both studies used similar arguments. This is because, and this is the key element, similar dynamics unfolds in disparate systems due to the microscopic interactions among the constituents, heart or brain cells. The study of emergence of similar collective dynamics in very different complex systems is flourishing these days (Cowan et al., 1999). Let me clarify that I have chosen these two studies on the control of cardiac and brain activity because of the similarity in their approach, which exemplifies the Dynamiceutical paradigm. This text is not meant to be a review on closed and open loop treatments, but as it turns out, the proposed dynamiceutical paradigm most likely will always results in a closed-loop, or on-demand, protocol.

These observations in the control of brain and cardiac activity offer an illustration of the advantages of theoretically-guided experimental strategies to abort pathological rhythms. If we ignore the analytical results and try to control epileptiform activity using, instead of the aforementioned dynamically-guided closed-loop method to stop seizure generation, an open-loop protocol—which is the normal paradigm in most of clinical DBS applications (but see Morrell, 2011)—we will find that same stimulation protocol (5 seconds at 5 Hz) reduces seizure frequency by 17%, compared with the ~90% reduction using the closed-loop system (Salam et al., 2016). It is fair to note that other studies using periodic pacing without a closed-loop approach found greater success rate at stopping seizures, even though in these cases the stimulation was applied for longer times (Barbarosie and Avoli, 1997; Panuccio et al., 2013). The reason that such a short stimulation in the previous studies (5 seconds) was efficient at preventing seizure generation is probably due to the typical extreme sensitivity to perturbations when a system is close to a dynamical bifurcation, as was elaborated in our publications. Such a short precisely-timed stimulus has the added advantage of these closed-loop methods in that they deliver less energy than conventional open-loop stimulation (Salam et al., 2016; Cagnan et al., 2017).

My contention is that investigating in depth the individual's physiological dynamics using a framework based on dynamical and complex system theory is the best suited to guide the perturbation of the pathological activities. The reason to focus on this framework is clear: the combined activity among the many systems and organs in the body is complex and dynamic, the resultant physiological rhythms emerging from the intricate web of interactions. The examples above have used linear stability theory to assess features of the fixed points of the dynamics, but other methods can be equally applied to unravel dynamical regimes; particularly, finding dynamical bifurcations may be of importance (Perez Velazquez et al., 2003). The advantage of identifying fixed points is that these represent the skeleton of the system's dynamics. Underlying these notions of fixed points (attractors) is the controversy about whether the attractor paradigm in biological activity is meaningful. There is no space to discuss these matters here as they have been treated in other texts (Perez Velazquez, 2005; Perez Velazquez and Frantseva, 2011). Suffice to say that while it is mostly transients and not real attractors what we find in physiological data, the continuous transitions between those transient dynamical regimes are still worth characterizing. Another potential problem is to find the variable, or rather, the pertinent order parameter that best captures the dynamics in each pathological condition. But none of these difficulties are unsurmountable.

## And what about machine learning?

It is clear from the discussions above that this approach is grounded in physics: predominantly complex and dynamical system theory. At this point, readers familiar with the exploitation of machine learning algorithms to classify, detect or correct physiological phenomena may be thinking whether there is any substantial difference with the dynamiceutical approach. Only a cursory look at engineering journals reveals the tremendous surge in machine learning applied to biological problems, from identifying novel genes (Chen et al., 2015) to communicating with the paralyzed (Chaudhary et al., 2017). However, the manner in which machine learning algorithms operate makes it very difficult to ascertain how the physiological alterations are really achieved. It may be true that the nature of the dynamics of the physiological aspect the algorithm is assessing remains relatively known, as these algorithms use a variety of features to accomplish a task, but those features are not easily accessible to us for an understanding of the underlying physiology. Thus we still remain ignorant as to what it does and what side-effects a particular, say, brain stimulation or schedule of a medication may cause. As an example taken from our own investigations on detecting precursors of epileptic seizures, we have worked with algorithms that handled up to about 90 features. The physiological representation of those feature combinations that resulted in successful detection of brain paroxysmal activity remained obscure to us, even though certain feature selection was applied. On the other hand, performing a detailed scrutiny of the dynamics under consideration reveals the (neuro)physiologically relevant parameters one can use to modify/control a pathological state, and facilitates the knowledge about the (neuro)physiological events that result after stimulation.

For example, let's look at how the “simultaneous perturbation stochastic approximation” (SPSA) would operate if it were to be applied to the control of brain activity (it has in effect been applied to alter *in vitro* activity in brain slices Panuccio et al., 2013). The SPSA is an optimization strategy that uses a gradient approximation of an objective function, ensuring the convergence to an optimal solution. The cost function could be represented by the physiological state derived from multivariate signals that are recorded; using training data sets, the SPSA algorithm “decides” what stimulation parameters have to be changed from one pulse to the next in order to abolish the abnormal brain rhythms. Hence, regardless of the potential success of this algorithm, it will be difficult to exactly know in neurophysiological terms what it is doing. The chances to succeed may be greater if the underlying physiology is known in detail, rather than letting an algorithm work it out on its own. At least, if we know the dynamics we can have indications as to what is being changed and what results are expected. Nevertheless, these algorithms may be successful if the dynamics of the pathology in question are not too complex. Perhaps this is the reason why an elaborate model-based computational evolution to optimize stimulation patterns in Parkinson's performed with equal efficacy at eliminating symptoms to the standard constant high-frequency stimulation (Brocker et al., 2017). While not trivial, the abnormal brain activity associated with Parkinson's disease is better characterized than that of other diseases like epilepsy or other neuropsychiatric syndromes that are targets for DBS (DeLong and Wichmann, 2012), in that the brain areas and oscillations involved in Parkinson's are much less variable between individuals than those areas and synchronization patterns found in the different epilepsies. Hence, there could be no need for a detailed scrutiny of its dynamics in order to design efficient protocols to stop the Parkinsonian rhythms. At the same time, it is true that the application of machine learning to personalized medicine is not intended to shed more light on the pathophysiology of the target disease, but rather to aid in the quest of novel interventions when knowledge is limited. As such, my words here should not be taken as implying that I do not support this approach, rather I am just stating the fact that it is a different one. In simple words: in machine learning it is the machine/algorithm that “learns”; in Dynamiceuticals, it is us who learn and instruct the machine what to do. In fact, there is the possibility that in the future the new algorithms could teach us and be able to describe the dynamical regimes that are being spotted and altered; if this comes to be true, machine learning and the dynamiceutical approach will be one common paradigm.

And still, after having claimed that the dynamiceutical scheme relies on the identification of the dynamics associated with a specific pathophysiology, this does not mean that the molecular/cellular physiology underlying the dynamics is characterized. A good example is the aforementioned control of epileptic activity. The high level of description used in those studies does not allow for a clear interpretation of the cellular mechanism that results in the control of seizures, even though some educated guess may be advanced—for instance, the low frequency stimulation may promote short-time synaptic depression, or other possible factors. Another example of the high-level description without lower-level details is the implementation of phase-specific stimulation to control essential tremor (Cagnan et al., 2017).

## Comparable approaches

There is nothing really new under the sun. So, is this Dynamiceutical concept really novel? Considering the popularity that personalized medicine is receiving in current times and the various reports commenting the need for customizing neurostimulation technology (for DBS mainly), it is to some extent surprising that few schemes have been advanced as to the manner in which this customized approach can be best resolved. There have been proposals that are closely related to the idea of the dynamiceutical approach, but these tend to be grounded on technology and not much emphasis is placed on the theoretical/analytical investigations. For instance, the ACP-based approach combines artificial, computational and parallel methods in intelligent systems and technology for integrative and predictive medicine (Wang and Wong, 2013). A neuroinformatics approach called the Virtual Brain models individualized brain activity, linking macroscopic and microscopic levels, and, the authors claim, this modeling provides “biologically interpretable data” (Falcon et al., 2016). Other texts provide a system neurosciences view on brain dynamics and its possible relation to neurofeedback (Medeiros and Moraes, 2014; Ros et al., 2014), views that are close to the dynamiceutical perspective. The data-driven computational model of Muldoon et al. (2016) is consistent with the dynamiceutical approach. Still, many other articles review the emerging field of neuromodulation (Kringelbach et al., 2007; Temel and Jahanshahi, 2015), but without emphasizing the approaches that can be taken to investigate the best procedures to perform such perturbations of brain activity. Hence, as occurs in almost any aspect of scientific research, the ideas are already floating there, and it is a matter of a small step to crystallize those approaches into this new field.

## Opportunities for industry-academia collaborations

The serious implementation of Dynamiceuticals will further integrate the relation between industry and academia and expand their pursuits. It is commonplace today the collaborations between companies, engineers and academics; the entrepreneurial interest in implantable devices, the lucrative possibilities of biotechnology, the commercialization of basic research…All these aspects favor the close association between researchers and entrepreneurs. Still, the immense majority of approaches to either neurostimulation or administration of medical treatments follows the aforementioned blind trial-and-error strategy without a rational informed approach grounded in the physiological dynamics of the systems to be treated. One can therefore envisage a company of the future where these teams are fully integrated. And if one adds the current enthusiasm with “Big Data” and thus the need to extract meaning of all those vast datasets, the emergence of institutions/companies with effectively integrated teams is not only very probable but also a necessity. Big Data, a trend that has appeared recently in several disciplines, refers to extremely large data sets that cannot be analyzed/processed with conventional analytical methods. Physiology, and neuroscience in particular, has many levels of analysis from synapses to systems, thus it can be fragmented by a lack of collaboration between investigators at all levels. There are already big budget consortia, initiatives that make a concerted effort to unify the field by bringing together the entire range of experts and research tools. This approach will rely especially on the success of “Open science,” that requires greater collaboration between disjointed research hubs. Perhaps the integration of all the knowledge will give rise to other novel fields like Dynamogenetics (using the term someone proposed); as Pharmacogenetics is the study of genetic differences in drug metabolic pathways affecting individual responses to drugs, Dynamogenetics could be the assessment of genotypes that give different responses to attempts at changing/normalizing systems' dynamics using drug or electrical therapies.

## Conclusion

The vision of Dynamiceuticals, the theoretically-guided medical therapies based on the exploration of the range of dynamical regimes of physiological variables, has important consequences for translational medicine. First and foremost, it can help unify fragmentary results already known in human physiology and complex and dynamical system theories, and create a common framework for the development of precise and efficient protocols to “reorganize” the diseased system. The physics approach in which it is based already exists, but the challenge will be to promote the coordination of teams composed of scientists, engineers and healthcare practitioners that can work together efficaciously. Whereas this text has emphasized neurostimulation, it can be applied to almost any pathology where variables can be recorded as time series. The benefits of the dynamiceutical approach are thus double, in terms of acquiring basic novel knowledge, at a relatively high level of description, about the dynamics of a pathophysiology and in terms of the practical application of that knowledge. While the dynamics are studied at a high-level of description that, as it stands now in this type of analysis, precludes the identification of the lower-level molecular/cellular events, this should not prevent us from advancing these approaches in the control of pathological activities.

## Author contributions

The author confirms being the sole contributor of this work and approved it for publication.

### Conflict of interest statement

The author declares that the research was conducted in the absence of any commercial or financial relationships that could be construed as a potential conflict of interest.
